# Canadian critical care nurses experiences on the front lines of the COVID-19 pandemic: a qualitative descriptive study

**DOI:** 10.1186/s12912-022-01105-8

**Published:** 2022-11-29

**Authors:** Kathleen Gamble, Srinivas Murthy, Sarah L. Silverberg, Nina Gobat, Lisa M. Puchalski Ritchie

**Affiliations:** 1 Li Ka Shing Knowledge Institute, Knowledge Translation Program, St. Michael’s Hospital, 30 Bond Street, Toronto, ON M5B 1W8 Canada; 2 Department of Pediatrics, BC Children’s Hospital, University of British Columbia, 4500 Oak Street, Vancouver, BC V6H 3V4 Canada; 3 Nuffield Department of Primary Care Health Sciences, University of Oxford, Oxford, OX2 6GG UK; 4 Department of Medicine, University of Toronto, Toronto, ON Canada; 5 Department of Emergency Medicine, University Health Network, Toronto, ON Canada; 6 Institute of Health Policy, Management and Evaluation, University of Toronto, Toronto, ON Canada

**Keywords:** COVID-19, Nurses, Pandemic preparedness, Pandemic response, Burnout, Moral distress

## Abstract

**Background:**

Recent pandemics have provided important lessons to inform planning for public health emergencies. Despite these lessons, gaps in implementation during the COVID-19 pandemic are evident. Additionally, research to inform interventions to support the needs of front-line nurses during a prolonged pandemic are lacking. We aimed to gain an understanding of critical care nurses’ perspectives of the ongoing pandemic, including their opinions of their organization and governments response to the pandemic, to inform interventions to improve the response to the current and future pandemics.

**Methods:**

This sub-study is part of a cross-sectional online survey distributed to Canadian critical care nurses at two time points during the pandemic (March–May 2020; April–May 2021). We employed a qualitative descriptive design comprised of three open-ended questions to provide an opportunity for participants to share perspectives not specifically addressed in the main survey. Responses were analyzed using conventional content analysis.

**Results:**

One hundred nine of the 168 (64.9%) participants in the second survey responded to the open-ended questions. While perspectives about effectiveness of both their organization’s and the government’s responses to the pandemic were mixed, most noted that inconsistent and unclear communication made it difficult to trust the information provided. Several participants who had worked during previous pandemics noted that their organization’s COVID-19 response failed to incorporate lessons from these past experiences. Many respondents reported high levels of burnout and moral distress that negatively affected both their professional and personal lives. Despite these experiences, several respondents noted that support from co-workers had helped them to cope with the stress and challenges.

**Conclusion:**

One year into the pandemic, critical care nurses’ lived experiences continue to reflect previously identified challenges and opportunities for improvement in pandemic preparedness and response. These findings suggest that lessons from the current and prior pandemics have been inadequately considered in the COVID-19 response. Incorporation of these perspectives into interventions to improve the health system response, and support the needs of critical care nurses is essential to fostering a resilient health workforce. Research to understand the experience of other front-line workers and to learn from more and less successful interventions, and leaders, is needed.

## Background

As of April 2022, approximately3.59 million Canadians have contracted COVID-19 and just over 38 095 have died from the disease [1]. Additionally, since June 2021 the number of COVID-19 infections in Canadian health workers (HWs) has increased from 94, 873 to 150, 546 (as of January 14, 2022) [2], which represents 4.2% of all COVID-19 infections across the country [2]. COVID-19 is not Canada’s first experience with a pandemic. In recent decades, outbreaks of infectious diseases, such as Severe Acute Respiratory Syndrome in 2003 [3], and H1N1 (swine flu) [4] in 2009, have caused various degrees of strain to the Canadian health care system [5].

Although shorter than the ongoing COVID-19 pandemic, these incidents provided a number of lessons in how to plan and prepare for future pandemics [3, 6]. Maunder, for example, notes that Severe Acute Respiratory Syndrome (SARS) demonstrated the impact that an uncontrolled outbreak of an infectious disease could have on the physical and mental health of Canadian HWs, especially those working in a hospital settings [3]. Not only were HWs more likely to contract SARS while working in hospitals during the outbreak, they were also more likely to develop psychological distress from their experience [3]. The SARS outbreak demonstrated the need to develop practical interventions that supported HWs’ emotional and mental well-being during and after such an experience, and that these interventions should consider how individuals respond differently to stress [3].

Despite these findings, studies of HWs’ experiences during the H1N1pandemic yielded similar results. For example, Mitchell et al.’s study [4] on the experiences of HWs during the H1N1 outbreak in 2009, found that strong communication strategies were key to ensuring the proper use of personal protective equipment among HWs in emergency departments [4]. Hodge et al.'s review of qualitative studies of Canadian HWs lived experiences during the H1N1 pandemic, found that strong communication strategies are also a way to support the emotional/mental health of HWs during a pandemic [7]. By ensuring adequate access to required resources (both human and material supplies), and preparing for issues such as moral distress and burnout among HWs, decision makers can “ensure the response to the next pandemic is even more effective” [7]. When planning for future pandemics the unique perspectives and experiences of Canadian HWs should be included as they offer valuable insights to what is needed to support a strong front line response to a pandemic.

Again, despite these findings, the Canadian response to COVID-19 indicates that these lessons from previous outbreaks were not fully considered [5, 8, 9], with gaps in implementation evident during the current pandemic. Additionally, research to inform interventions to better support front-line nurses, and critical care nurses in particular, during a prolonged pandemic are lacking. Over the past two years, HWs have encountered a number of growing workplace stressors that affect both their professional and personal lives [5, 8–10]. As Silverberg et al. note, HWs play a crucial role in providing quality health care for patients with COVID-19 [11]. However, as the pandemic has progressed, HWs have faced numerous challenges, including higher patient volumes, a higher assumption of personal risk of contracting COVID-19, the limited availability of resources (such as personal protective equipment) and increased rates of burnout [5, 8–10, 12].

In addition, while HWs are recognized to play a key role in the ongoing response to COVID-19, several studies have found that their unique perspectives and experiences have often been largely overlooked [5, 6, 10]. Since 2021, this gap in knowledge has started to be addressed by an increasing number of qualitative studies describing the experiences of nurses during the COVID-19 pandemic [13]. This research has started to highlight how issues like excessive workloads, misinformation/miscommunication, a shortage of personal protective equipment (PPE), and a lack of support from health care leadership/management have placed enormous pressure on those working on the frontlines [13].

However, knowledge is still lacking around whether or not nurses in all fields/specialties are experiencing the impact of the COVID-19 pandemic in similar (or different) ways, with critical care nurses likely to experience unique challenges. For example, critical care nurses are often at an increased risk of COVID-19 infection as they spend more time directly caring for critically ill patients, and are exposed to higher risk procedures (i.e. aerosol generating procedures) that require reliable access to PPE specific to this higher risk setting. In addition, the complex needs of critical care patients and their families may be particularly difficult to meet within the context of the both human and material resource shortages experienced, and infection control restrictions encountered during the pandemic, which can increase both the physical and psychological/emotional toll on nurses in this setting. By identifying unique challenges faced by critical care nurses during this pandemic, policy makers and health care researchers can design interventions and strategies tailored to support the unique needs of critical nurses on the front lines of future pandemics.

### Study aim

This manuscript reports the findings of a sub-study, which is part of larger study aimed at evaluating nurses’ readiness to follow infection prevention and control guidelines in the workplace, and to understand their perceptions of trust in organizational preparedness, communication, and their perceptions of personal risk. The aim of this sub-study was to gain an understanding of critical care nurses’ perspectives of the ongoing COVID-19 pandemic based on their lived experiences, including their opinions on how their organization and various levels of government responded to the pandemic. The overall goal of the sub-study is to inform interventions to improve the Canadian health care systems response to both the current and future pandemics, including interventions to support the unique needs of critical care nurses.

## Methods

### Study design

This sub-study is part of a larger study that used a cross-sectional online survey to assess the perspectives of Canadian HWs at two points during the COVID-19 pandemic. A detailed description of the original survey's development and findings was previously published [11]. The original survey evaluated respondents’ readiness to follow infection prevention and control guidelines in their workplace and to understand their perceptions of trust in organizational preparedness, communication, and infection risk during the first wave of the pandemic [11]. It was adapted for the Canadian context from an international survey developed by the World Health Organization’s COVID-19 Research Roadmap Social Science and IPC workings groups at the beginning of the pandemic [11]. The main survey contained a total of 41 Likert scale questions and 13 targeted questions about participants’ demographic details and work context [11]. The original survey was distributed between March 16, 2020 and May 25, 2020, during the first wave of COVID-19 in Canada [11]. The survey was distributed a second time between April 29, 2021 and May 28, 2021 to assess for change overtime with minor changes to the main survey [14]. In order to gain an understanding of the lived experience of critical care nurses on the front lines of the pandemic, we used this opportunity to add a sub-study that employed a qualitative descriptive design comprised of three optional open-ended questions (Table 1), that were included as part of the second survey distribution.

**Table 1:** Open-Ended Survey Questions

1	What do you think was done well by your organization and/or the government in responding to the COVID pandemic?
2	What would you like to see done differently by your organization and/or the government in response to future epidemics/pandemics?
3	Is there anything about your experience working clinically during the COVID pandemic that you would like to share?

The sub-study questions were purposely designed to provide an opportunity for participants to elaborate on questions from the larger survey, and to allow for the emergence of perspectives of importance to participants that were not specifically addressed in the main survey. Open-ended questions capture data that cannot be obtained using quantitative surveys alone [15, 16]. Open-ended questions have the potential to reveal what respondents think of spontaneously while they are filling out the survey, and are not biased by provided response options [17].

#### Survey distribution

For the second distribution, we employed convenience sampling via the email distribution list and slack channels of the Canadian Association of Critical Care Nurses. This is a large national network of critical care nurses (1100 list serve members), which was felt to be representative of nurses in a variety of Canadian critical care settings [11].

The Association is a volunteer organization with the goal of promoting quality patient care for Canadian’s experiencing life threatening illness and injury [18]. Its membership includes registered nurses, nursing students, and allied health professionals who have interests in the science of critical illness care. The Canadian Association of Critical Care Nurses is overseen by a National Board of Directors with representatives from Eastern, Central and Western parts of Canada. There are approximately 1400 active members of the Canadian Nurses Association [18].

#### Data collection

The surveys were self-administered digitally via Research Electronic Data Capture [19]. Data for the second distribution, including this sub-study, was collected between April 29 and May 28, 2021, with three reminders sent out. The survey remained open for an additional two weeks following the final reminder.

#### Data analysis

Responses to the sub-study questions were analyzed using conventional content analysis [20]. Conventional content analysis involves the development of codes directly from the data and is generally used when a study’s aim is to describe a phenomenon, or in this case the lived experiences of Canadian critical care nurses during the COVID-19 pandemic [20]. Unlike a directed approach, this type of content analysis does not start with a theory or relevant research findings as guidance for initial codes [20]. This more inductive approach was chosen to allow for emergence of themes through a less biased lens.

NVivo 12 was used to code and organize the data. Two study team members (KG, LPR) independently coded the data in two rounds. KG is a non-clinician researcher, who has taught extensively in undergraduate Nursing and Health Policy programs at three Ontario universities. LPR is an emergency physician and implementation scientist based in large urban academic hospitals in Ontario. After the completion of the first round, each team member’s list of codes were shared and discussed so that a consensus could be reached and a coding framework developed before the second round of analysis. Once this framework was created, the same two-team members used it to conduct a second round of data analysis. Themes were sought across individuals with consideration of age, gender, years of experience, and hospital type (academic, community).

#### Techniques employed to enhance rigour and trustworthiness

Several methods were employed during data collection and analysis to enhance the rigor of this study’s findings. First, as part of the survey introduction, participants were informed that their responses to the three open-ended questions were optional, and that their answers would remain anonymous. Additionally, no identifying data was collected in the survey. Second, data source and analyst triangulation were employed [21]. Data source triangulation examines data from a variety of sources (participants) within the same data collection technique. In this study, data was gathered from a wide range of respondents who represent different experiences of critical care nurses across Canada, allowing for examination of consistency across the range of data sources. Analyst triangulation was employed in the analysis of participants’ response to the sub-study questions. This involves the use of more than one analyst in a study and the ability to compare and contrast findings among analysts without prior discussion or collaboration between analysts and enhances the credibility of the findings. Triangulation was undertaken in 2 steps, with convergence and divergence of themes examined first across data sources and then across analysts. Third, notes were taken throughout coding independently and collaboratively throughout the analysis process, to provide an audit trail and allow for reflexivity [22]. Finally, exemplar quotes are provided throughout the results and in Table 3 to highlight representative quotes for themes and sub-themes [22].

#### Ethical approval and informed consent

The study received ethical approval from the University of British Columbia /Children’s and Women’s Health Centre of British Columbia Research Ethics Board in Vancouver, BC (Reference number: H20-00,803).

## Results

Participant characteristics are provided in Table 2. Themes and subthemes with example quotes are provided in Table 3.

**Table 2:** Participant Characteristics

Participant Characteristics	Mean	Range
**Age**	41 years	25 to 60 years
**Length of Service**	17 years	2 to 41 years
	**Number**	**Percentage**
**Gender**		
Female	90/109	82.3%
Male	12/109	11.0%
Non Binary	1/109	0.92%
Prefer Not Say	5/109	0.46%
No Answer	1/109	0.92%
**Location**		
BC	9/109	8.3%
AB	19/109	17.4%
SK	4/109	3.7%
NVT	1/109	0.92%
MAN	6/109	5.5%
ON	49/109	44.9%
QUE	8/109	7.3%
NB	3/109	2.8%
PEI	0/109	0.0%
NS	7/109	6.4%
NFLD	2/109	1.8%
No Answer	1/109	0.92%
**Place of Employment**		
Community Hospital	44/109	40.4%
Academic Hospital	63/109	57.8%
Other	1/109	0.92%
No Answer	1/109	0.92%
**Employment Status**		
Full Time	81/109	74.3%
Part Time	19/109	17.4%
Casual	8/109	7.4%
No Answer	1/09	0.92%
**Job Role**		
Bedside Nurse	90/109	82.3%
Charge Nurse	16/109	14.7%
Other (E.g. Nurse Educator)	2/109	1.8%
No Answer	1/109	0.92%
**Provide Direct Care to Patients**		
Yes	105/109	96.3%
No	3/109	2.8%
No Answer	1/109	0.92%
**Previous Experience Working during an epidemic/pandemic (e.g. SARS, MERS, H1N1)**		
Yes	73/109	66.9%
No	35/109	32.1%
Unsure	1/109	0.92%
**Personally Cared for Patients with suspected/confirmed infection caused by respiratory pathogen (e.g. SARS, MERS, H1N1)**		
Yes	85/109	77.9%
No	22/109	20.2%
Unsure	2/109	1.8%
**Personally cared for Patients with suspected/confirmed COVID-19 infection**		
Yes	105/109	96.3%
No	4/109	3.7%

**Table 3:** Themes and Sub-themes with Example Quotes

Theme	Sub-Theme	Example Quotes
Team Work		“Most of our staff keep showing up and volunteering to fill holes in the schedule. The efforts of our staff have made all the difference in the world.” “Senior nurses have supported me fully and I have gained great insight and experience” "It is the teamwork, it is the colleagues, and it is the work relationships that have been so incredibly stable during this time. I have learned new coping skills and strengths that I didn't even know I had. This pandemic has forced me to become a stronger and better RN.”
Resource Shortages	Material Resource Shortages	“I am still having trouble obtaining adequate N95 supply. I am forced to use/re-use the same mask for the whole 12 h shift in the ICU.” “Ensure adequate oxygen supply at all hospitals to support surge of covid patients—Should have been recognized earlier”
	Staff Shortages	“Our covid unit was understaffed, patients suffered tremendously because of this. If anything was traumatic, it was the poor care that was given due to lack of staff, untrained staff and educators, as well as absent intervention by our management team to improve conditions for isolated patients.” “The current situation makes me feel unprepared to care for my patients in the ICU. We often have 2–3 patients per ICU nurse with non ICU staff assisting. This becomes a problem try to manage very acute patients with multiple infusions, and provide even basic care like line changes and prone positioning”
	Staff Redeployment	“It was good to see how fast the nursing community came together to help further outbreaks by having nurses come out of retirement and management roles to help. Great job to all redeployed staff.” “I am stretched to the limit working with inexperienced poorly trained "extenders" and exhausted experienced nurses who are asked to support them” “Altering the scope of nurses to staff units is also difficult as you are dependent on experienced staff to train the new staff and this is not a great environment for teaching/learning”
Organizational and Government Preparedness & Policy	Organizational level	“I have worked through the aids crisis SARS MERS H1N1 and now this. From my experience very little was learned in the previous crisis situations. We are again flying by the seat of our pants, the expectations for front line staff are unclear, and even when they are clear no one in management takes responsibility to ensure the expectations are met.” “The illness and deaths are one thing but there is so much more suffering being created not only by the disease but by policies and planning that seems misguided or ineffective.” “More policy to guide decision making. Currently we are told rules and it’s up to staff to determine how they wish to implement the rules, and there is nothing in place to guide decisions.” “From an organizational perspective, we were given the appropriate PPE and received training on respirator usage. I felt supported by the organization I was working with. I was also able to receive my vaccine very quickly through my organization.”
	Government Level	“Federal and Provincial governments, along with local hospitals need to develop a coordinated plan should this happen again. Everything from PPE stockpiling and distribution to informing visitation policies in hospital. Most of the time it felt like nurses on the ground were left making up rules as we went along because there was no effective leadership from anyone.” “Our province was woefully under prepared for this pandemic, despite having time & warning to prepare. It has highlighted how severely short staffed the nursing profession is as a whole, and it has put many practitioners in unsafe situations regarding their practice “The Federal government failed in closing the borders soon enough to travelers…” “The provincial government has often failed to be consistent, trying to save lives and the economy simultaneously.”
Leadership & Communication	Leadership	“The expectations for front line staff are unclear, and even when they are clear no one in management takes responsibility to ensure the expectations are met.” "The entire thing has felt like we (bedside nurses) were left alone to figure out how to care for people and how to protect ourselves. Very little effective leadership came from my institution or the government. Policies changed constantly but no effort was made to ensure the latest changes were known by people on the ground." “My organization has great leadership, centralization of supplies including PPE, cohorting Covid patients, frequently updating us and answering questions in a weekly town hall.”
	Communication	“When directives are being changed almost daily, it leads to lack of trust in the system. Recognizing that knowledge on a virus changes frequently and protocols are adapted to meet the growing body of knowledge, it must equally be recognized that making frequent changes can be unsettling to bedside staff and develop a sense of dishonesty and lack of trust.” “Better communication about policies and procedures. I work mostly night shifts and there are no members of the leadership team available to inform decision making when new situations arise” “Our unit managers were honest with us every step along the way, recognizing errors that were made and good decisions that were taken. Every week, they told us what the plan was for the following week” “Consistency of direction from Public health and Government. Avoid confusing messaging. Would have been nice if provincially and federally there was consistency. Should not be Political.”
Impact on Staff	Burnout	“This has been the most stressful year of my professional practice. I've been constantly inundated with all things COVID. There has been nowhere for me to escape it- personally and professionally. This has led to a significant amount of burnout- not only for me personally but for many of my coworkers as well.”
	Moral Distress	“In 30 years, I have never had this amount of moral distress caused by lack of insight by our management team. The daily suffering is not as distressing as the lack of intervention and support for our patients who are suffering because they can't get a bath or assistance with their meals”
	Personal Safety and Impact on Family	“This is been personally a hard on. I have been an ICU nurse for 3 years. I worked the entire wave 1 + 2. At the begin[ning] of wave 3 I became pregnant. So navigating safety for me and my baby has been hard.”
	Impact of Public Opinion	“It is exhausting to continuously see and hear members of the public saying that masks don't work and restrictions don't work and that the pandemic is a hoax when every shift we see physical evidence of the reverse of all those statements “The apathy of the public towards the severity of the health risks and the need for precautions is shocking. …. The public really doesn't seem to understand or care and it has lead me to not want to be a nurse anymore.”
	Professionalism & Work Ethic	“I am devastated by the lack of compassion and work ethic in the young nurses coming into our professional.” “Not all of us are on the same page. Our allied health staff are not supporting nurses at all, shy away from work and nurses ending up doing everyone’s job”
	Staff Wellness Resources	“There isn’t appropriate supports in place for staff wellness as this pandemic rages on…the [current] wellness program [was] created [with] strategies that they thought would benefit people without asking us what we need” “I am tired of this lock down emotionally and physically tired. We need more emotional support for the workers inside hospitals that have had to watch people die every day from something could have been prevented.”
	Staff Appreciation	"Listen to front line staff because we can actually explain what is happening and what we need." "The recognition that a nurse is not a nurse. They are specialists in their roles." “I would like pandemic pay to be carried through the entire pandemic for health care staff. It is a small way of [showing] appreciation.”

### Respondent characteristics

Of the 168 nurses who participated in the second round of the survey, 109 (64.9%) provided written responses to the open-ended questions and were included in the sub-study. The majority of the 109 respondents self-identified as female (82.3%), and the mean age of the participants was 41 years. On average, respondents have been practicing for 17 years (with a range of 2 years to 41 years) and provided direct patient care (96.3%) while working full time in academic hospitals (57.8%). The majority of respondents were from Ontario (44.9%), with a large number of responses from Alberta (17.4%) and British Columbia (8.3%). 66.9% of participants had previously worked in a clinical setting during an epidemic, and 77.9% had personal experience caring for patients with a suspected or confirmed infection caused by a novel respiratory pathogen (i.e. SARS, COVID-19, H1N1). Most respondents (96.3%) had personally cared for a patient with COVID-19.

As the majority of participants identified as female, and worked in academic/community hospitals, we could not assess if/how gender or hospital type may have influenced the experience of working during the pandemic. Differences based on participant age and past experience working in a pandemic are included below in the summary of results under each theme.

### Teamwork

Despite the obvious challenges and stresses caused by COVID-19, some participants reported positive experiences and outcomes of the pandemic. In particular, support and encouragement from their fellow coworkers were noted to help in coping with the stresses and challenges of providing clinical care in the context of the pandemic. As one participant stated, “most of our staff keep showing up and volunteering to fill holes in the schedule. The efforts of our staff have made all the difference in the world.” Likewise, another respondent wrote, “about 50% of frontline workers are going above and beyond to work extra hours and are giving all they can to care for patients."  As one participant stated, in midst of so much uncertainty one of the only things that remained consistent was the efforts of her fellow nurses:

"It is the teamwork, it is the colleagues, it is the work relationships that have been so incredibly stable during this time. I have learned new coping skills and strengths that I didn’t even know I had. This pandemic has forced me to become a stronger and better RN."

Overall, the majority of the respondents felt that their co-workers consistently “went above and beyond what is normally expected” and described this as an ongoing positive experience that encouraged many of the participants to carry on despite deteriorating situations that included significant shortages in resources.

### Resource shortages

Numerous participants described shortages of both material and human resources, and challenges as a result of redeployment to address staff shortages.

#### Material resource shortages

A majority of respondents expressed frustration and anger over the lack of personal protective equipment (PPE) they received at work. As one respondent noted, “I am still having trouble obtaining adequate N95 supply. I am forced to use/re-use the same mask for the whole 12 h shift in the ICU (intensive care unit)”. Another respondent wrote, [we] “are expected to wear the same mask in and out of COVID rooms for the duration of that period…this prolonged wear [is] uncomfortable and sometimes the mask seal is sketchy after several hours”. In addition to PPE shortages, participants also noted shortages in medical supplies, including inadequate supplies of oxygen during COVID surges, a lack of bed space for patients, and improperly ventilated hospital rooms.

#### Staff shortages

Severe staffing shortages were noted to be an ongoing and constant concern throughout the pandemic for most respondents. Staffing shortages worsened over the course of the pandemic due to illness and issues around retention, and the redeployment of nurses to address staffing shortages in critical areas. As one respondent noted, it was the lack of human resources more so than material resources that made it difficult to manage high volumes of patients during surges in the pandemic:

"We need higher baseline capacity of ICU beds and staff. We learned quickly that physical space and equipment were not the bottleneck. It was experienced ICU nurses."

As well, many respondents felt there were several times when “nurses [ended] up doing everyone’s job” and had “to pick up slack…when they were already stretched too thin”. This left many participants feeling overwhelmed as reported below in the ‘Impact on Staff’ section of the results. In order to address critical staffing shortages, many organizations redeployed their staff into different roles.

#### Staff redeployment

Participants’ perspectives about redeployment to different positions within the hospital were generally negative and were usually tied to views about the quality of training received by redeployed staff. As one respondent shared, their organization did not “have transparent information on how people are being redeployed and how it [was] happening”. Likewise, another participant said, “I’m a PICU RN forcefully redeployed to bedside care in adults. It has been an experience, little to no training”. Some respondents also expressed frustration about having to work with redeployed staff who did not know how to work in specific areas, or as one respondent shared, “I am stretched to the limit working with inexperienced poorly trained ‘extenders’”. Likewise, another participant stated, “redeployed nurses: some do bedside [care] and some do not (so they just walk around and not "help")”.

### Organizational and government preparedness & policy

Subthemes under this theme focus on preparedness to respond and actual response to the pandemic with respect to public health policy and guidance at both the government and organization levels.

Respondents’ perspectives about the effectiveness of both their organization’s and the government’s ongoing response to the pandemic were mixed. As one respondent stated, “I think we have been lagging behind in the pandemic in both organization and governmental response”. Another participant wrote, “nothing has changed in my facilities’ approach to this pandemic… [we] did not implement any rules or guidance after H1N1″. In addition, eight respondents specifically noted that "nothing" or "not enough" was done by the government and/or their organization. On the other hand, another participant shared that their hospital has done well [by] providing staff with regular education…ample PPE… [and] frequent organizational updates on the status of our response”.

#### Government level

Many participants agreed that the government’s response was inadequate. As one participant shared, the “government [responded] too slowly to rising cases [and] is too passive with enforcement of restrictions” and another said, “the provincial and federal governments need to listen to experts in [the] field… [they] should have kept lockdown in Jan 2021 longer… [governments] too worried about businesses”. While the majority of respondents shared negative views on this point, some noted that “the government, both provincial and federal, sought out the best advice available” and others wrote that their provincial government “had done an incredible job in coordinating limited provincial resources to relieve hospitals…done an amazing job in promoting and organizing COVID 19 testing”.

#### Organizational level

Respondents also had mixed views with respect to organizational pandemic preparedness and policy. While some felt that their “hospital[s] had done well… [in] providing staff with regular education on prevention and control and…and frequent organizational updates”, others found their organization’s response to be “very unorganized” and found it “hard to think of anything they've done well". As one respondent shared, “the constant changing policies made a difficult situation worse” as it left them wanting a “better sense of transparency” from their organization’s leadership teams.

Several participants, who shared that they had worked during previous infectious outbreaks (i.e. SARS and H1N1), expressed concern when it seemed like their organization’s pandemic policy had not taken into account lessons learned from these previous experiences:

"I have worked through the AIDS crisis, SARS, MERS, H1N1 and now this…from my experience very little was learned in the previous crisis situations”.

Some respondents attributed this to a lack of pre-planning and a failure to “implement any [new] rules or guidance after H1N1 [which] leads to last minute and dangerous decisions” in the midst of an ongoing crisis.

### Leadership & communication

This theme focused on leadership at both the government and organizational level, with effectiveness of communication frequently noted as a specific aspect of leadership.

Participants’ opinions about the quality of government leadership were generally critical. As one respondent stated, “I don’t believe our government (provincially or federally) have handled this pandemic well. They have lacked transparency and clarity, which has only enhanced public mistrust and has negatively impacted people’s willingness to comply with public health measures”. Some participants also felt that government decision makers did not listen to advice from health care professionals:

"The provincial government…refused to listen to the health care professionals and scientists …... NO ONE asked for or sought out critical care nursing opinion. There is a GREAT divide between federal and provincial health care responsibilities. Communication between the two is very sadly lacking."

Participant views of hospital leadership and communication were mixed. Some participants expressed positive views of the leadership and communication within their hospitals by saying “I think [managers] are doing their best. We have support with PPE for code blues, management and upper leadership tries to make themselves available to hear about issues”. Likewise, “our unit managers were honest with us every step along the way, recognizing errors that were made and good decisions that were taken. Every week, they told us what the plan was for the following week”. However, other respondents shared views critical of hospital leadership: “We are flying by the seat of our pants, the expectations for front line staff are unclear, and even when they are clear no one in management takes responsibility to ensure the expectations are met”. One participant wrote, “I have never been as disappointed and saddened by the response from nursing executives and nursing managers as I am during this pandemic”.

Although somewhat mixed, one of the most common critiques that participants had about both their organization’s response and the effectiveness of leadership at all levels (i.e. within the hospital and from the government), focused on the quality and frequency with which they received information. While one respondent noted that, “the hospital tried to keep staff up to date on the ever changing rules regarding COVID-19”, others noted a variety of problems with communication. As another participant stated, “when directives are being changed almost daily, it leads to lack of trust in the system. Recognizing that knowledge on a virus changes frequently and protocols are adapted to meet the growing body of knowledge, it must equally be recognized that making frequent changes can be unsettling to bedside staff and develop a sense of dishonesty and lack of trust”. Miscommunication occurred at multiple levels according to respondents. Several pointed out that unclear messaging at both the organization and government levels made it difficult to trust the information being shared at all levels.

### Impact on staff

Subthemes under this theme reflect specific categories of participants’ personal and professional life noted to have been impacted by the pandemic. While categories overlap or occur together in some cases, subthemes are titled to reflect relatively distinct categories to facilitate understanding.

For many participants, working on the front lines of the pandemic has “been exhausting” and several reported feeling “stretched to the limit”. Many respondents shared this perspective, with several noting that the toll the pandemic has had on nurses was largely overlooked by both their organization and various levels of government. Or, as one participant stated, “I don’t feel like sharing anymore because no one listens”. While there was some variation in how participants described these feelings, many responses reflected strong feelings of ‘burnout’ and ‘moral distress’.

#### Burnout

A number of respondents noted ‘burnout’ to be a common issue among nursing staff. As one participant wrote, “staff is being burnt out fast”. Many respondents felt like they “received very little support from [their] management teams for the sacrifices [they have] made to care for the sickest of the sick” and at times made them feel like “they were doing everyone’s job [and] were stretched too thin”.

There were several respondents who made note of the “need [for] more outlets to help with the mental health of staff” and some also shared that a loss of a loved one to COVID-19 amplified these feelings. There was a shared perspective among many respondents that “the long-term effects of this pandemic will be felt for years to come…[and] we need a strategy to address the mental health toll this pandemic is taking on our staff”.

#### Moral distress

Several participants noted experiencing substantial feelings of moral distress caused by patient suffering and/or loss, and their inability to provide optimal care. As one respondent shared,

"the current situation makes me feel unprepared to care for my patients in the ICU. We often have 2-3 patients per ICU nurse…in the event of an acute deterioration you are really stretched for ICU level help…if more than one patient deteriorates it is traumatizing to staff who are unable to get any assistance."

Another respondent described distress due to the understaffing of COVID units, which caused patients to suffer tremendously because of “the poor care that was given due to lack of staff, untrained staff…as well as absent intervention[s] by management team[s] to improve conditions for isolated patients”.

Many respondents also shared how they watched their patients suffer alone and that it was “extremely difficult [to interact] with families when they are unable to be at the bedside of critically ill patients”. This added to feelings of moral distress for many participants because “illness and deaths are one thing but [when] so much more suffering [is] created not only by disease but by policies and planning that seem misguided or ineffective” it becomes overwhelming and impacts how people do their jobs.

#### Personal safety and impact on family

A number of respondents shared that both working and living through the pandemic has also taken a significant toll on their personal well-being and sense of safety:

"This has been the most stressful year of my professional practice. I’ve been constantly inundated with all things COVID. There has been nowhere for me to escape it-personally or professionally."

Several participants also described how they “received very little support from [their] management teams for the many sacrifices [they’ve] made” and have “contemplated leaving [their] career”. Or, as one respondent shared “it's been very scary & challenging…the hardest time…within…my 25 years as an RN”.

Participants also worried about the safety of their loved ones and described the burden of carrying the extra “stress of infecting family members”. Some also described the day-to-day struggle they had figuring out how to manage things like “childcare when spouse[s] also work shift work and [were] unable to work from home”. As well, several respondents talked about their personal safety when sharing their experiences of being pregnant during the pandemic and described it as “very stressful… [especially] with little research on the vaccines” impact on pregnancies.

#### Impact of public opinion

Negative public opinions about nurses had a significant impact on a number of our respondents, and greatly affected their sense of well-being and at times their personal safety as well. As one participant shared:

"I have noticed an ongoing lack of care for nurses. I hide the fact that I am a nurse as people react poorly in many cases. Even friends have made comments…that indicate that they feel I am a cesspool of the COVID-19 virus."

This sense of frustration was shared by a number respondents who were angry to “see and hear members of the public [continuously] saying that masks don’t work and restrictions don’t work and that the pandemic is a hoax”, when “every shift [they] see physical evidence of the reverse of all those statements”. The majority of people outside of hospitals “have very little idea of what is going on behind the hospital doors” and there was consensus among participants that if more people understood the realities of the pandemic “more people would follow the public health guidelines and…give real support to the healthcare workers”.

#### Professionalism & work ethic

Lack of professionalism or work ethic among some HWs was a theme noted principally by senior nurses, (i.e. nurses who had been practicing for several years vs. nurses who were newly graduated or early on in their careers). As one participant stated, “as a critical care nurse in a tertiary hospital, we are doing everyone’s job…just about everyone is abusing us and taking advantage of us”. Likewise, another participant said, “our allied health staff are not supporting nurses at all…none of us are on the same page”. Several respondents expressed dismay over “the lack of compassion and work ethic in young nurses coming into [the] profession”.

In some cases, respondents indicated that this lack of professionalism was also a failure in morality as “it is morally wrong to come to work to do nothing” and in other cases participants noted that it was because other health care professionals were scared of being exposed to the virus:

"We emptied the hampers because hospital assistants do not come into “isolation rooms…respiratory techs ask nurses to adjust vents (ventilators) settings because they would not want to ‘expose’ themselves. It means nurses have to pick up the slack".

#### Staff wellness resources

Some respondents noted that better resources for Staff Wellness would be beneficial. As one participant stated, “there isn’t appropriate supports in place for staff wellness as this pandemic rages on…the [current] wellness program [was] created [with] strategies that they thought would benefit people without asking us what we need”. Or, as one participant shared, “creating a program without consulting what trained professionals need to support them” will just mean the program is ineffective.

#### Staff appreciation

Several participants also expressed interest in receiving financial compensation for their efforts or talked about the way financial incentives could be used to offset the increased burden they experienced while providing care during the pandemic. As one participant wrote, “I would like pandemic pay to be carried through the entire pandemic for health care staff. It is a small way of [showing] appreciation”. Higher pay wages for nurses may also keep them in their jobs”. Some of these opinions were driven by the belief that by putting themselves at risk and “working in stressful riskier environments” they needed the incentive and support to “feel better about getting up everyday to risk [themselves] and [their] families for others”. Others expressed feeling that their knowledge and expertise were not recognized or valued, as one participant noted [there is a lack of] "The recognition that a nurse is not a nurse. They are specialists in their roles".

## Discussion

Our sub-study of critical care nurses’ perspectives 1 year into the COVID-19 pandemic highlight important insights from the unique lived experiences of individuals working on the front lines of this ongoing crisis. Overall, respondents reported that working in critical care during a pandemic was highly stressful and that these feelings of stress were amplified when respondents did not feel supported by their organization and/or government. In our study, the majority of respondents noted that inconsistent and unclear communication had a significant impact on their personal sense of well-being and while our study captured a variety of views, participants’ perspectives on these points were generally quite critical.

Our findings in this sub-study, with respect to perceptions of organizational preparedness and communication, align with the results from round two of this survey and suggests there has been a shift in critical care nurses perspectives from round one of the survey conducted in 2020 [14]. Silverberg et al. findings on the first survey indicated that early on in the pandemic, critical care nurses had a significant amount of trust in the healthcare system and generally felt that they had some autonomy and control over whether they contracted COVID-19 [11]. As well, while many of the nurses surveyed in this first round of research expressed a strong concern for personal health and risk of exposure to family, they also felt empowered and in control of their own situation because of the comfort level, they had around their knowledge and use of personal protective equipment [11].

However, responses to the 2021 survey showed a significant drop in perceptions of the readiness, honesty, and trust in institutions to act in the best interests of citizens at the level of region and national governments [14]. Our sub-study supports this view with some respondents reporting dissatisfaction with their organization’s and government’s response to the pandemic and increasing frustration with the lack of clear communication at both the organizational (i.e. hospital) and governmental level.

Our findings are consistent with previous research that defines burnout as the “emotional exhaustion, depersonalization, and [a] diminished [sense] of professional achievement” [3, 23], with many participants reporting experiencing distressing levels of burnout that have negatively affected both their professional and personal lives. In addition, our findings align with previous research that found working on the frontlines of a pandemic makes nurses more susceptible to this experience, especially when they feel unsupported or under supported by decision-makers, organizational leaders, and/or government officials [5, 6, 12, 23].

Previous research has also shown that pandemics can have a significant psychological, physical, and emotional impact on nurses [5, 8, 10, 12, 23, 24]. As Fernandez notes, research from both the ongoing COVID-19 pandemic and previous pandemics demonstrate the need to develop policies that support the emotional, mental and physical well-being of nurses working on the frontlines during these emergencies [5].Psychological support, received during and after a pandemic, can be a key factor in reducing these feelings of burnout among nurses [23, 25, 26]. These findings are also reflected in the responses of our participants, which note a lack of control in their workplace and lack of support from managers and leadership teams within their organization, and substantial feelings of physical, emotional and professional distress.

Although there is an increasing number of studies detailing the experiences of nurses during the pandemic, there are few focused on the specific perspectives of critical care nurses. As Rhéaume et al. note, specific research is needed in this area because critical care nurses often face a number of challenges that are unique to their field of practice [13]. Examples of these challenges include higher rates of patient-related issues that cause moral distress, higher rates of burnout and a higher risk of infection as they spend more time directly caring for COVID-19 patients [13]. The type of care critical nurses provide is highly stressful and as the pandemic has progressed, nurses in this field have been expected to provide complex care to severely ill patients in environments that change rapidly, and where there is often little reliable information being shared [13].

Similarly, a study by Moradi et al. also found that the challenges faced by critical care nurses are unique and directly related to the type of care they provide patients [27]. This study highlighted how issues related to excessive workloads, a shortage of PPE, and unreliable/unclear communication around changing pandemic policies, all contributed to participants feeling unsupported and undervalued by their organizations [27]. Even prior to the pandemic, critical care nurses had the highest rates of burnout among different nursing specialities [27], and what the studies by Rhéaume and Moradi illustrate is that the COVID-19 pandemic has amplified all these issues to a point of crisis.

Our research adds to this body of knowledge by providing critical care nurses in this study an opportunity to describe their lived experiences of caring for patients during the pandemic. Many of the experiences of our participants in this study were similar to those shared by participants in the studies by Rhéaume and Moradi. Like the critical care nurses in these other studies, the respondents is this survey also described feelings of moral distress, burnout, and being unsupported by the organization. As a result, our research will also contribute to a better understanding of the unique challenges critical care nurses face and demonstrate why interventions that are specifically tailored to the experiences of critical care nurses need to be developed to bolster the Canadian health care system’s response to both the current and future pandemic.

### Study strengths and limitations

Strengths of this sub-study include the use of an open-ended question format and the timing of the second survey distribution. Open-ended questions provide an opportunity for respondents to expand/elaborate on their perspectives and to introduce concepts not assessed through standard closed-ended survey questions. This allows for a broader understanding of nurses’ lived experience of working on the front lines of a pandemic. In addition, distributing the survey approximately 1 year into the pandemic provided an opportunity to assess the lived experience of nurses working in the unique context of a prolonged pandemic.

Our sub-study also has several limitations. Overall, the response rate to the survey was low. As well, most of the respondents were critical care nurses working in academic hospitals, which may not reflect the experience non-critical care nurses or those working in different settings. Finally, as overwhelming majority of our respondents self-identified as female, we were unable to assess whether gender played a role in respondents’ perspectives.

## Conclusion

Our-sub study found that approximately 1 year into the COVID-19 pandemic, although perspectives were mixed in some areas, the lived experiences of critical care nurses continue to reflect previously identified challenges and opportunities for improvement. In particular, challenges with organizational and government preparedness, response, leadership, and communication, were frequently noted, as were failure to adequately recognize the contributions of, and support the health and wellness needs of front line workers.

Although the prolonged nature of the COVID-19 pandemic has placed an unprecedented challenge on the Canadian health care system, the experiences shared by the participants in this survey suggest that lessons from early in the current pandemic, and those from prior pandemics, have been inadequately incorporated into interventions to lessen the impact on front line HWs and critical care nurses in particular. Future pandemic preparedness needs to incorporate these perspectives to foster a resilient critical care health workforce.

Future work to compare and contrast more and less successful sites, interventions and leaders, and to understand the experiences of other front line HWs, including those working outside critical care in other high burden health care settings such as long-term care, is needed to inform current and future interventions.

## Data Availability

The data that support the findings of this study are available on reasonable request from the authors, at srinivas.murthy@cw.bc.ca. The data are not publicly available due to them containing information that could compromise research participant privacy/consent.

## Abbreviations

HWs: Heath Workers
COVID-19: Coronavirus disease
SARS: Severe acute respiratory syndrome
H1N1: Subtype influenza A virus
MERS: Middle East respiratory syndrome
PPE: Personal protective equipment
ICU: Intensive care unit
AIDS: Acquired immunodeficiency syndrome

## References

[1] Canada Go. Public Health Services Coronavirus Disease (COVID-19) Current Situation 2022. https://www.canada.ca/en/public-health/services/diseases/coronavirus-disease-covid-19.html. Available from

[2] Information CIfH. COVID-19 Cases and Deaths in Health Care Workers in Canada 2022. https://www.cihi.ca/en/covid-19-cases-and-deaths-in-health-care-workers-in-canada. Available from

[3] Maunder R (2004). The experience of the 2003 SARS outbreak as a traumatic stress among frontline healthcare workers in Toronto: lessons learned. Philos Trans R Soc Lond B Biol Sci.

[4] Mitchell R, Ogunremi T, Astrakianakis G, Bryce E, Gervais R, Gravel D (2012). Impact of the 2009 influenza A (H1N1) pandemic on Canadian health care workers: a survey on vaccination, illness, absenteeism, and personal protective equipment. Am J Infect Control.

[5] Fernandez R, Lord H, Halcomb E, Moxham L, Middleton R, Alananzeh I (2020). Implications for COVID-19: a systematic review of nurses' experiences of working in acute care hospital settings during a respiratory pandemic. Int J Nurs Stud..

[6] Naylor D, Basrur S, Bergeron M, Brunham R, Butler-Jones D, Dafoe G, Ferguson-Pare M, Lussing F, McGeer A, Neufeld K, Plummer F. Learning from SARS: Renewal of Public Health in Canada. A report of the National Advisory Committee on SARS and Public Health October 2003. Health Canada. 2003. Available at: https://chrome-extension://efaidnbmnnnibpcajpcglclefindmkaj/ https://www.canada.ca/content/dam/phac-aspc/migration/phac-aspc/publicat/sars-sras/pdf/sars-e.pdf.

[7] Hodge J (2014). Canadian Healthcare Workers' Experiences During Pandemic H1N1 influenza: Lessons from Canada's Response.

[8] Combden S, Forward A, Sarkar A (2022). COVID-19 pandemic responses of Canada and United States in first 6 months: a comparative analysis. Int J Health Plann Manage.

[9] Baskin RG, Bartlett R (2021). Healthcare worker resilience during the COVID-19 pandemic: An integrative review. J Nurs Manag.

[10] Havaei F, Smith P, Oudyk J, Potter GG (2021). The impact of the COVID-19 pandemic on mental health of nurses in British Columbia, Canada using trends analysis across three time points. Ann Epidemiol.

[11] Silverberg SL, Puchalski Ritchie LM, Gobat N, Murthy S (2021). COVID-19 infection prevention and control procedures and institutional trust: perceptions of Canadian intensive care and emergency department nurses. Can J Anaesth.

[12] Shaukat N, Ali DM, Razzak J (2020). Physical and mental health impacts of COVID-19 on healthcare workers: a scoping review. Int J Emerg Med.

[13] Rhéaume A, Breau M, Boudreau S (2022). A critical incident study of ICU nurses during the COVID-19 pandemic. Nurs Ethics.

[14] Silverberg SL, Puchalski Ritchie, Lisa M., Gobat, Nina, Murthy, Srinivas. Canadian Intensive Care Nurses' Infection Prevention and Control Adherence and Institutional Trust: An Update 1-year into the Pandemic. Can J Crit Care Nurs. 2022;33(2): Fall 2022. https://cjccn.ca/featuredarticle/canadian-intensive-care-nurses-infection-prevention-and-control-adherence-and-institutional-trust-an-update-1-year-into-the-pandemic/.

[15] Riiskjær E, Ammentorp J, Kofoed P-E (2012). The value of open-ended questions in surveys on patient experience: number of comments and perceived usefulness from a hospital perspective. Int J Qual Health Care.

[16] Marcinowicz L, Chlabicz S, Grebowski R (2007). Open-ended questions in surveys of patients' satisfaction with family doctors. J Health Serv Res Policy.

[17] Brosius A, Hameleers M, van der Meer TGLA. Can we trust measures of trust? a comparison of results from open and closed questions. Qual Quant. 2022;56(5):2907–24. 10.1007/s11135-021-01250-3 PMC8493540 34629555

[18] Nurses CAoCC. About CACCN 2022. https://caccn.ca/about-caccn/. Available from

[19] Harris PA, Taylor R, Thielke R, Payne J, Gonzalez N, Conde JG (2009). Research electronic data capture (REDCap)—a metadata-driven methodology and workflow process for providing translational research informatics support. J Biomed Inform.

[20] Hsieh H-F, Shannon SE (2005). Three approaches to qualitative content analysis. Qual Health Res.

[21] Patton MQ (1999). Enhancing the quality and credibility of qualitative analysis. Health Serv Res.

[22] Squires A, Dorsen C (2018). Qualitative research in nursing and health professions regulation. J Nurs Regul.

[23] Manzano García G, Ayala Calvo JC (2021). The threat of COVID-19 and its influence on nursing staff burnout. J Adv Nurs.

[24] Yu F, Raphael D, Mackay L, Smith M, King A (2019). Personal and work-related factors associated with nurse resilience: a systematic review. Int J Nurs Stud.

[25] Balicer RD, Omer SB, Barnett DJ, Everly GS (2006). Local public health workers' perceptions toward responding to an influenza pandemic. BMC Public Health.

[26] Corley A, Hammond NE, Fraser JF (2010). The experiences of health care workers employed in an Australian intensive care unit during the H1N1 Influenza pandemic of 2009: a phenomenological study. Int J Nurs Stud.

[27] Moradi Y, Baghaei R, Hosseingholipour K, Mollazadeh F (2021). Challenges experienced by ICU nurses throughout the provision of care for COVID-19 patients: A qualitative study. J Nurs Manag.

